# Lobe-Based Hepatic Uptake Index of Gd-EOB-DTPA on Contrast-Enhanced MRI to Quantitatively Discriminate between Compensated and Decompensated Hepatitis B-Related Cirrhosis

**DOI:** 10.1155/2024/6623848

**Published:** 2024-06-21

**Authors:** Fulin Lu, Bangguo Tan, Yucheng Huang, Lin Xu, Changqiang Wu, Haiying Zhou, Rui Li, Xiaoming Zhang, Tianwu Chen, Hongjun Li

**Affiliations:** ^1^Department of Radiology, Sichuan Academy of Medical Sciences and Sichuan Provincial People's Hospital, University of Electronic Science and Technology of China, Chengdu 610072, Sichuan, China; ^2^Department of Radiology, Panzhihua Central Hospital, Panzhihua 617067, Sichuan, China; ^3^Department of Radiology, The Third Hospital of Chengdu, Chengdu 610031, Sichuan, China; ^4^Department of Radiology, Dazhou Center Hospital, Dazhou 635099, Sichuan, China; ^5^Medical Imaging Key Laboratory of Sichuan Province and School of Medical Imaging, Affiliated Hospital of North Sichuan Medical College, Nanchong 637000, China; ^6^Department of Radiology, The Second Affiliated Hospital of Chongqing Medical University, Chongqing 400010, China; ^7^Department of Radiology, Beijing YouAn Hospital, Capital Medical University, Beijing 100069, China

## Abstract

**Purpose:**

To use hepatic uptake index (HUI) of liver lobes on gadolinium ethoxybenzyl diethylenetriamine pentaacetic acid (Gd-EOB-DTPA)-enhanced magnetic resonance imaging (MRI) to discriminate between patients with hepatitis B-related cirrhosis in compensated and decompensated statuses.

**Methods:**

Forty-four consecutive patients with hepatitis B-related cirrhosis who underwent Gd-EOB-DTPA-enhanced MRI were divided into compensated and decompensated statuses based on clinical evaluation. Volume and signal intensity of individual lobes were retrospectively measured to calculate HUI of the right liver lobe (RHUI), medial (MHUI) and lateral (LHUI) left liver lobes, and caudate lobe (CHUI). Spearman's rank correlation analyses were performed to evaluate relationships of lobe-based HUI with Child–Pugh and model for end-stage liver disease (MELD) scoring system scores in compensated and decompensated statuses. The Mann–Whitney U-test was used to compare the lobe-based HUI between compensated and decompensated statuses. The performance of lobe-based HUI in distinguishing cirrhosis was evaluated using receiver operating characteristic (ROC) analysis, and the area under the ROC curve (AUC) was calculated as a measure of accuracy. Delong's method was used for statistical analysis to elucidate which HUI is optimal.

**Results:**

Compensated and decompensated liver cirrhosis were confirmed in 25 (56.82%) and 19 (43.18%) patients, respectively. According to Spearman's rank correlation analysis, RHUI, MHUI, LHUI, and CHUI were all significantly associated with Child–Pugh and MELD scores (all *P* values <0.05). Receiver operating characteristic analysis demonstrated that among all lobe-based HUI parameters, RHUI could best perform the previous discrimination with a cut-off of 485.73 and obtain an AUC of 0.867. The AUC of RHUI improved and was significantly different from that of MHUI, LHUI, and CHUI (*P* = 0.03, *P* = 0.007, and *P* < 0.001, respectively, Delong's test).

**Conclusions:**

The RHUI could help quantitatively discriminate hepatitis B-related cirrhosis between compensated and decompensated statuses.

## 1. Introduction

Cirrhosis caused by hepatitis B is commonly observed in many Asian countries, leading to increased morbidity and mortality rates [1]. Liver cirrhosis is the advanced stage of liver fibrosis, and it causes chronic damage to major vital organs, such as the spleen, brain, and digestive tract, and results in bad outcomes [2]. Within clinical settings, cirrhosis is categorized into two states: compensated and decompensated. This categorization is based on the presence of symptoms such as gastroesophageal variceal bleeding or ascites [3]. Different clinical treatments are required for patients with compensated and decompensated liver cirrhosis, as their survival rates significantly differ [4]. Liver function evaluation plays a crucial role in monitoring and preoperative assessment of patients with liver cirrhosis, especially to determine cirrhosis patients in compensated or decompensated status [5]. In clinical practice, several methods such as the biochemistry measurement of aspartate transferase and alanine transferase, clinical grading systems based on the Child–Pugh stages and the model for end-stage liver disease scoring systems, and the dynamic quantitative tests based on the indocyanine green (ICG) clearance test have been used to evaluate the liver function [6]. However, all these methods focus on evaluating overall liver function and do not specifically assess individual lobe-based liver function [7].

Gadolinium ethoxybenzyl diethylenetriamine pentaacetic acid (Gd-EOB-DTPA) is a paramagnetic hepatobiliary magnetic resonance contrast agent. Due to its OATP_1_B_1_/B_3_-dependent hepatocyte-specific uptake system and paramagnetic properties, increasing evidence has emerged to suggest that Gd-EOB-DTPA-enhanced MRI could be potentially used for evaluation of liver function [8]. Twenty minutes after administration of Gd-EOB-DTPA, approximately 50% of the injected dose is transferred into the hepatocytes and gets eventually excreted into the bile in healthy human liver [9]. The clearance of Gd-EOB-DTPA is reliant on the functional integrity of the hepatocytes, and reduced liver uptake of Gd-EOB-DTPA during the hepatobiliary phase (HBP) magnetic resonance imaging typically indicates impaired liver function [10].

Lobe-based liver function assessment is crucial for evaluating surgical risk, particularly for surgeons aiming to predict remnant liver function following partial hepatectomy [11, 12]. However, previous studies mostly focused on signal intensity (SI) changes on Gd-EOB-DTPA-enhanced MR images in cirrhotic patients and did not take the volume change of liver lobes into consideration [13, 14]. To the best of our knowledge, there are no reports that have utilized both SI and volume changes in liver lobes to evaluate the lobe-based function in patients with hepatitis B-related cirrhosis between compensated and decompensated statuses. Therefore, we have combined the volume and signal intensity on Gd-EOB-DTPA-enhanced MR images to calculate the hepatic uptake index (HUI) of individual liver lobes and use HUI to discriminate the function of liver lobes between compensated and decompensated statuses in patients with hepatitis B-related cirrhosis.

## 2. Materials and Methods

### 2.1. Patients

The study protocol underwent a thorough review and received approval from the Institutional Review Board of our hospital (NSMC-2021-81). The retrospective study adhered to the institutional guidelines for consent waiver as individual consent was not required. The study was performed entirely according to the basic principles of the Declaration of Helsinki.

From January 2017 to December 2020, forty-nine patients with confirmed hepatitis B-related cirrhosis who underwent Gd-EOB-DTPA-enhanced MR imaging at our institution were continuously collected in this study. Patients were enrolled in this study according to the following inclusion criteria: (a) diagnosis of hepatitis B-related cirrhosis based on characteristic findings from radiological examinations, laboratory tests, and physical findings, following the clinical practice guidelines established by the American Association for the Study of Liver Diseases (AASLD) involving chronic hepatitis B (2015) [15]; (b) liver cirrhosis was diagnosed based on clinical manifestations, imaging examinations, laboratory tests, and histopathological examination. Determination of compensated and decompensated cirrhotic statuses within one week after hospital admission by using a wildly accepted classification system as proposed by D'Amico: compensated status (absence of esophageal varices and ascites, or presence of nonbleeding esophageal varices without ascites, hepatic encephalopathy, and hepatorenal syndrome), and decompensated status (presence or current presence of a second decompensation event including ascites, variceal bleeding, jaundice, or hepatic encephalopathy). Patients with bleeding alone who developed ascites, jaundice, or hepatic encephalopathy after bleeding esophageal varices recovered [16], and (c) triple phase (including arterial, venous, and delayed phases) and 20-min hepatobiliary phase (HBP) Gd-EOB-DTPA-enhanced abdominal magnetic resonance (MR) scans were performed on the cirrhotic patients within one week after admission. Exclusion criteria were as follows: (a) patients associated with hepatic interspace occupation lesions such as hepatocellular carcinoma (*n* = 2); (b) patients with liver cirrhosis who had ever intra-abdominal surgery (*n* = 2); (c) incomplete examination and dissatisfactory quality of the MR images (*n* = 1); or (d) patients with esophageal variceal bleeding that were not caused by esophageal varices but by other etiologies such as ulcerative gastrointestinal bleeding (*n* = 0). Ultimately, our study included a total of forty-four patients (29 men and 15 women; mean age, 54.20 ± 11.06 years, and range, 24–69 years). Also, the cirrhotic patients were divided into compensated status in 25 (56.82%) and decompensated status in 19 (43.18%).  Table 1 records the clinical data of the patients, including their sex, age, presence or absence of esophageal variceal bleeding, and ascites.

### 2.2. MR Imaging

All examinations were performed using a 3.0 T MRI scanner (Discovery MR 750, GE Medical Systems, Milwaukee, WI, USA) with a 32-channel body phased-array coil. The conventional unenhanced MRI scans included the three-dimension liver acquisition with volume acceleration flexible (3D-LAVA-FLEX) T_1_-weighted imaging and the axial fast spoiled gradient echo T_2_^*∗*^-weighted imaging (SPGR T_2_^*∗*^WI). Enhanced axial triple-phase and HBP scanning were performed using a breath hold axial LAVA-FLEX sequence with Gd-EOB-DTPA (Primovist, Bayer Schering Pharma AG, Berlin, Germany) as a hepatocellular MR contrast agent. For the contrast-enhanced scans, the contrast agent was intravenously administrated via a power injector at a rate of 1.0 ml/s with a total dose calculated according to 0.025 mmol/kg body weight, followed by a 20–25 mL saline flush. The HBP images were obtained 20 minutes after the intravenous administration of Gd-EOB-DTPA. The parameters of the axial triple-phase scanning were as follows: repetition time (TR) of 4.2 ms, echo time (TE) of 1.80 ms, number of excitations of 1.0, flip angle of 12°, field of view (FOV) of 352 × 400 mm, matrix of 268 × 236, reconstruction matrix of 400×400, bandwidth of 200 Hz per pixel, thickness of 5.2 mm, and thickness spacing of 0 mm. The parameters of the HBP scan were mostly the same as those of triple-phase scans except number of excitations of 2.0, flip angle of 15°, bandwidth of 166.67 Hz per pixel, and thickness of 3 mm.

### 2.3. Imaging Analysis

The MR data were presented on a picture archiving and communication system (PACS; GE Advantage Workstation Version 4.4-09, Sun Microsystems, Palo Alto, CA, USA) with an optimal window setting adjustment. As reported by Goldsmith and Woodburne [17], the liver could be divided into four lobes through the hepatic fissures and hepatic veins, including the right lobe, left medial and lateral lobes, and caudate lobe. Two radiologists, who were blind to the clinical data of the enrolled patients with 3 years (the first author) and 24 years (the corresponding author) of experience in diagnostic imaging, independently drew the outlines of the individual liver lobes to calculate the sectional area of the liver lobe on portal-venous phase images. The sectional areas of each liver lobe on each section were added together to calculate the total sectional area of each lobe. Ultimately, the total sectional area of the individual liver lobe was multiplied by the slice thickness to obtain the volume of each liver lobe.

**Figure 1:**
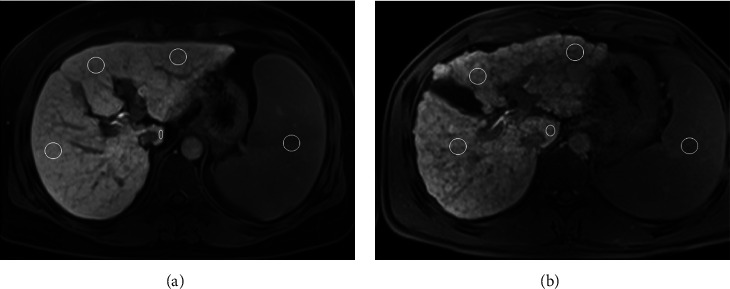
Two patients with hepatitis B-related cirrhosis in compensated (a) and decompensated (b) statuses have undergone gadolinium ethoxybenzyl diethylenetriamine pentaacetic acid-enhanced axial T_1_-weighted magnetic resonance imaging. Circles with areas of 1-2 cm^2^ on the 20 minutes hepatobiliary-phase magnetic resonance images are regions of interest used for the measurements of signal intensity of the right liver lobe, left medial liver lobe, left lateral liver lobe, caudate lobe, and spleen.

**Figure 2:**
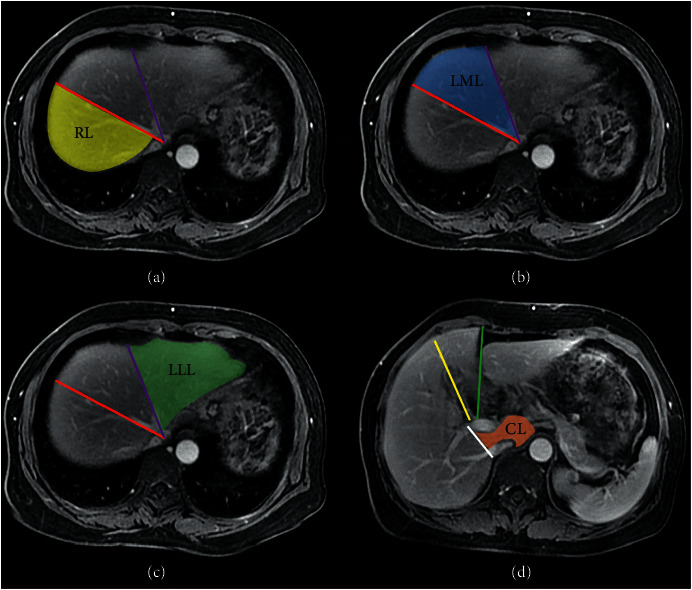
For the division of liver lobes on the axial-enhanced portal-venous phase magnetic resonance images, the middle liver vein (red line, (a)) is used as a landmark to differentiate the right liver lobe (RL) from the left liver lobe. The left liver vein (purple line) is used as a landmark to differentiate the left medial liver lobe (LML, (b)) from the left lateral liver lobe (LLL, (c)). The line linking the right branch of the portal vein to the inferior vena cava (white line) is used as a landmark to differentiate caudate lobe (CL) from RL (d).

To quantitatively measure the signal intensity (SI) of liver lobes and spleen, regions of interest (ROIs) were drawn on the 20 min HBP images. The SI values of each liver lobe and spleen were randomly measured in at least five consecutive sections that were evenly distributed. Each ROI was either a circle or an oval with defined area of 1-2 cm^2^ (Figure 1). In order to accurately measure the SI values of each liver lobes, we excluded extra hepatic structures including the biliary system, the gallbladder, portal veins, hepatic arteries and veins, and inferior vena cava (Figure 2) when drawing the ROIs within individual lobes. We also excluded the splenic artery and vein when drawing the ROIs of the spleen to measure its SI.

For calculating the uptake indexes of individual hepatic lobes including the HUI of the right lobe (RHUI), the left medial lobe (MHUI), the left lateral lobe (LHUI), and the caudate lobe (CHUI), we combined the individual liver lobe volume together with the average SI of the corresponding liver lobe and spleen to calculate the individual liver lobe HUI by using the [18] following formula:

(1) $$\begin{array}{c} \text{LV}\times \left(\frac{{\text{SI}}_{\text{ hepaticlobes}}}{{\text{SI}}_{\text{spleen}}}-1\right), \end{array}$$

where LV represented the volume of liver lobes, and SI_hepaticlobes_ and SI_spleen_ represented the SI values of the corresponding liver lobes and spleen, respectively. The results obtained by the two radiologists were used to test the interobserver agreement of the individual liver lobe HUI.

### 2.4. Statistical Analysis

All statistical analyses were performed by using commercial software (SPSS version 26.0, SPSS Inc., Chicago, IL). The level of statistical significance was set at *P* < 0.05.

In this study, the distribution types of RHUI, MHUI, LHUI, and CHUI tested by the Shapiro–Wilk analysis were conformed to the skew distribution and were expressed as median (25th–75th percentile). Interclass correlation coefficient (ICC) was used to assess the agreement of individual liver lobe HUI measurements between the two observers. ICC values less than 0.5, between 0.5 and 0.75, between 0.75 and 0.90, and greater than 0.90 represented poor, moderate, good, and excellent repeatability, respectively [19]. The correlation of individual liver lobe HUI with the Child–Pugh and MELD scores of liver cirrhosis was tested by Spearman's rank correlation analysis. Differences in median values of the RHUI, MHUI, and LHUI were tested by using the Mann–Whitney *U*-test. The receiver operating characteristic (ROC) analysis was used to explore sensitivity and specificity of identifying cirrhosis between compensated and decompensated statuses with RHUI, MHUI, LHUI, and CHUI. Delong's method was used for statistical comparison to elucidate which HUI parameter is optimal [20].

## 3. Results

### 3.1. Interobserver Agreement of HUI Measurements

The individual liver lobe HUI measurements were proven to be of excellent repeatability between the two radiologists, with ICC values of 0.96 (95% confidence interval (95%CI), 0.94–0.99) for RHUI, 0.91 (95%CI, 0.89–0.97) for MHUI, 0.93 (95%CI, 0.91–0.97) for LHUI, and 0.89 (95%CI, 0.86–0.92) for CHUI with all *P* values less than 0.001, respectively.

### 3.2. Individual Liver Lobe HUI: Compensated vs. Decompensated Statuses

**Figure 3:**
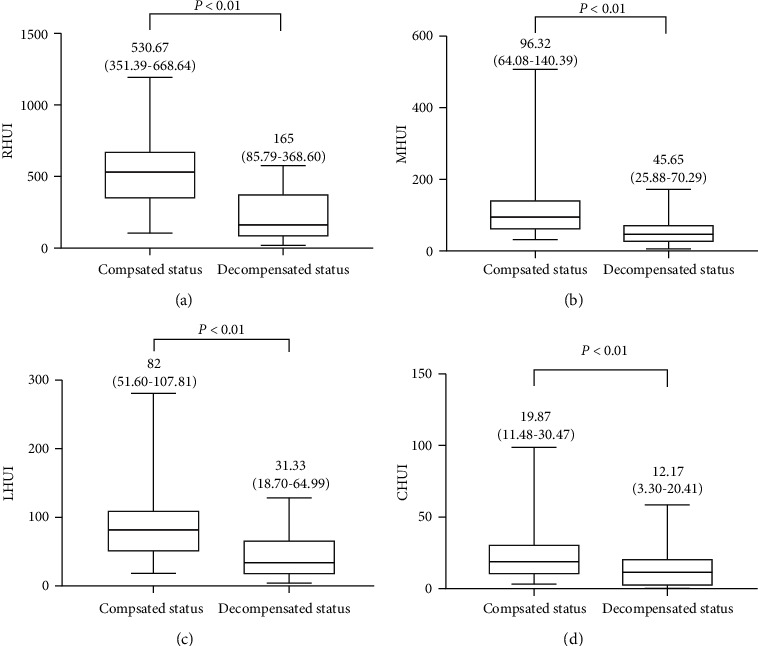
Mann–Whitney U-tests of hepatic uptake indexes of the right liver lobe (RHUI), left medial liver lobe (MHUI), left lateral liver lobe (LHUI), and caudate lobe (CHUI) with liver cirrhosis between compensated and decompensated statuses are shown in images (a), (b), (c), and (d), respectively.

As shown by the Mann–Whitney *U*-test, the RHUI, MHUI, LHUI, and CHUI were significantly higher in patients with compensated cirrhosis than with decompensated cirrhosis (Figure 3). The RHUI, MHUI, LHUI, and CHUI were negative correlations with compensated statuses as shown in Figure 3. The correlation between RHUI, MHUI, LHUI, and CHUI with Child–Pugh and MELD scores are shown in Table 2.

### 3.3. Discrimination between Compensated and Decompensated Cirrhosis by ROC Analysis

**Figure 4:**
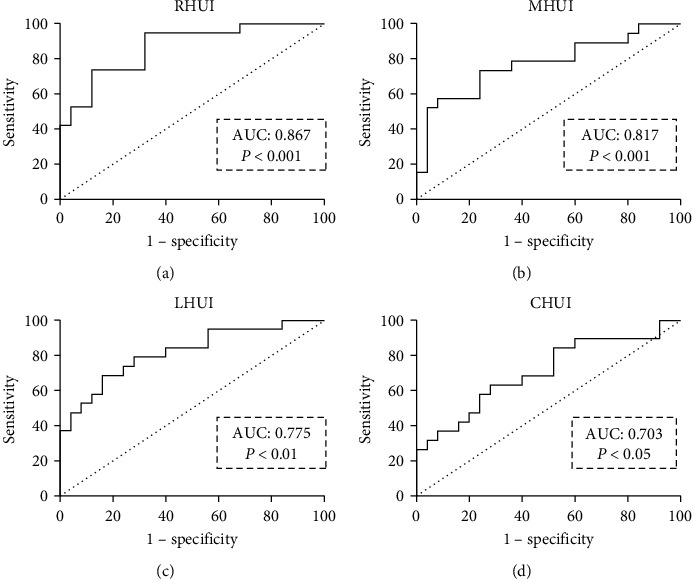
Receiver operating characteristics (ROC) curves of the hepatic uptake indexes of the right liver lobe (RHUI, a), left medial liver lobe (MHUI, b), left lateral liver lobe (LHUI, c), and caudate lobe (CHUI, d) are used to discriminate the hepatitis B-related cirrhotic patients between the compensated and decompensated statuses.

Based on the results of the Mann–Whitney *U*-test, ROC analysis of the RHUI, MHUI, LHUI, and CHUI (Table 3) were further performed to test the independent parameter to test the distinction sensitivity and specificity between compensated and decompensated statuses. The ROC analysis revealed that the optimal cut-off value of RHUI of 485.73 could obtain the highest area under the ROC curve (AUC) of 0.867 with a sensitivity of 68%, and a specificity of 94.7% to distinguish between patients with compensated and decompensated hepatitis B-related cirrhosis (Figure 4). The AUC of RHUI improved and was significantly different from that of MHUI, LHUI, and CHUI (*P*=0.03, *P*=0.007, and *P* < 0.001, respectively, Delong's test).

## 4. Discussion

It is widely recognized that the function of liver lobes will decrease as cirrhosis develops, especially in decompensated cirrhotic patients [21]. HUI was first used to access the entire liver function reported by Yamada et al. [18]. However, to date, no studies have investigated the possibility of liver lobe-based HUI to discriminate between compensated and decompensated liver cirrhosis. In this study, we innovatively explored the HUI of liver lobes measured on Gd-EOB-DTPA-enhanced HBP MR images to discriminate hepatitis B-related cirrhotic patients between compensated and decompensated statuses.

In our study, the liver lobe-based HUI has taken both the changes of signal intensity on Gd-EOB-DTPA-enhanced HBP MR images and the volume of individual liver lobes on portal-venous phase images into consideration. Previous studies showed that the severity of cirrhosis could significantly affect the ratio of the hepatocyte absorption of Gd-EOB-DTPA after administration, and cirrhotic patients would present a significant decrease of signal intensity in liver parenchyma on Gd-EOB-DTPA-enhanced HBP MR images [22, 23]. In addition, the morphology of liver lobes undergoes alterations as cirrhosis progresses [24, 25]. Histologically, the collapse of liver structures, pronounced distortion of hepatic vascular architecture, and diffuse nodular regeneration surrounded by dense fibrotic septa could not only lead to the signal intensity decrease on Gd-EOB-DTPA-enhanced HBP MR images but also lead to the volume reduction of hepatic lobes [26, 27]. Consequently, we utilized this composite biomarker, the HUI, to investigate whether this parameter of individual liver lobes could aid in distinguishing between compensated and decompensated statuses in hepatitis B-related cirrhosis. Our study revealed that the RHUI, MHUI, LHUI, and CHUI are higher in patients with compensated cirrhosis than with decompensated cirrhosis, and the RHUI showed the best correlation with compensated and decompensated statuses. This finding can be explained by three mechanisms. First, previous studies showed that the liver volume would decrease with the progression of liver cirrhosis due to the reduction in the number of normal hepatocytes and the blood flow from the portal venous [28, 29]. Furthermore, research studies have demonstrated that hypoproteinemia and portal hypertension could cause bowel wall thickening and liver-to-abdominal area ratio score decreasing [30, 31]. These research studies' results could also indirectly explain the decline of lobe-based HUI in decompensated liver cirrhosis patients. Second, the reduction of normal hepatocytes could also lead to a decreased absorptivity of Gd-EOB-DTPA, which can be manifested as the decreased signal intensity on HBP MR images, indicating that the previous signal intensity of the liver could decrease from compensated status to decompensated status. Third, liver cirrhosis causes a decrease in the extracellular fluid space of the spleen, which can be observed as a decrease in spleen enhancement, although the decrease may not be as significant as that in the liver, as demonstrated in an animal study [32]. All these factors could result in a decrease of HUI on Gd-EOB-DTPA-enhanced MR images when liver cirrhosis develops from the compensated status to decompensated status. For the right liver lobe, the reduction of normal hepatocytes and blood flow can be more significant than that for any other liver lobes, which can lead to a more significant decrease of RHUI in the decompensated status than in the compensated status [33, 34].

Our study aimed to investigate the potential of lobe-based HUI, which combines the signal intensity and volume of liver lobes, to differentiate between hepatitis B-related cirrhotic patients in the compensated and decompensated statuses. Our findings indicated that the RHUI could serve as an independent discriminator. The ROC analysis revealed that the RHUI has good discriminating performance to discriminate hepatitis B-related cirrhotic patients between compensated and decompensated statuses (AUC: 0.867, *P* < 0.05 Delong's test). Furthermore, our study demonstrated that the individual liver lobe-based HUI measurements could be of excellent repeatability, implying that the evaluation index we selected can be reliable for subsequent studies.

However, our study does have a limitation. The sample size was relatively small, but our study provided some useful information of the liver lobe-based HUI for hepatitis B-related cirrhotic patients. A large-scale study will be performed in our future study to confirm the results.

## 5. Conclusion

Our study creatively takes both the parenchyma signal intensity and the volume of individual liver lobes into consideration to explore the most suitable biomarker to discriminate between the compensated and decompensated statuses in hepatitis B-related cirrhotic patients. This study revealed that the RHUI could be a reliable parameter to perform the previous discrimination with an AUC of more than 0.85. We believe that our findings have the potential to assist in the quantitative determination of the compensated status in cirrhotic patients, enabling the selection of appropriate treatment strategies to delay the progression of liver cirrhosis and improve patient survival.

## Figures and Tables

**Table 1:** Clinical characteristics and liver lobe-based hepatic uptake index of the enrolled patients.

Variable	Total	Compensated status	Decompensated status	
Patients (%)	44 (100)	25 (56.82)	19 (43.18)	
Sex: male vs. female (no. of patients)	30 vs. 14	20 vs. 5	10 vs. 9	
Age (y)	53.8 ± 10.84	49.52 ± 10.74	59.42 ± 8.24	
Aspartate aminotransferase (IU/L)	57.89 ± 39.19	53.73 ± 37.33	64.73 ± 35.78	
Alanine aminotransferase (IU/L)	53.08 ± 38.24	50.60 ± 37.25	61.50 ± 39.14	
Total bilirubin (mg/dL)	1.09 ± 0.53	1.04 ± 0.49	1.13 ± 0.55	
Albumin (*μ*mol/L)	38.21 ± 5.04	39.67 ± 5.51	37.72 ± 5.31	
Ascites: yes vs. no (no. of patients)	16 vs. 25	0 vs. 25	16 vs. 0	
Gastroesophageal varices: with vs. without bleeding (no. of patients)	5 vs. 13	0 vs. 13	5 vs. 0	
Both ascites and gastroesophageal varices bleeding (no. of patients)	21	0	21	
Child–Pugh score		Stage A (5-6)	Stage B (7–9)	Stage C (≥10)
No. of patients	44	25	13	6
MELD score		Stage A (≤14)	Stage B (15–18)	Stage C (≥18)
No. of patients	44	25	14	5
Liver lobe-based HUI				
RHUI	368.3 (178.70–558)	530.67 (351.39–668.64)	165 (85.79–368.6)	
MHUI	72.45 (41.64–109.23)	96.32 (64.08–140.39)	45.65 (25.88–70.29)	
LHUI	60.73 (32.11–94.23)	82 (51.60–107.81)	31.33 (18.70–64.99)	
CHUI	18.23 (7.35–27.03)	19.87 (11.48–30.47)	12.17 (3.30–20.41)	

Notes: RHUI, hepatic uptake index of right liver lobe; MHUI, hepatic uptake index of left medial liver lobe; LHUI, hepatic uptake index of left lateral liver lobe; CHUI, hepatic uptake index of caudate lobe; MELD, model for end-stage liver disease.

**Table 2:** Correlations of individual liver lobe-based hepatic uptake index with Child–Pugh and MELD in hepatitis B-related cirrhosis.

	RHUI	*P*	MHUI	*P*	LHUI	*P*	CHUI	*P*
Child–Pugh	−0.734	<0.001	−0.631	<0.01	−0.571	0.012	−0.446	0.037
MELD	−0.652	0.025	−0.544	0.036	−0.539	0.039	−0.318	0.041

Notes: RHUI, hepatic uptake index of right liver lobe; MHUI, hepatic uptake index of left medial liver lobe; LHUI, hepatic uptake index of left lateral liver lobe; CHUI, hepatic uptake index of caudate lobe; MELD, model for end-stage liver disease.

**Table 3:** Receiver operating characteristic analysis of lobe-based hepatic uptake index to discriminate between compensated and decompensated statuses in hepatitis B-related cirrhosis.

Parameter	Cut-off value	AUC	95% CI of AUC	Sensitivity	Specificity
RHUI	485.73	0.867	0.761–0.973	0.68	0.947
MHUI	52.93	0.817	0.688–0.945	0.84	0.684
LHUI	39	0.775	0.629–0.92	0.92	0.579
CHUI	15.25	0.703	0.544–0.863	0.72	0.632

Notes: RHUI, hepatic uptake index of right liver lobe; MHUI, hepatic uptake index of left medial liver lobe; LHUI, hepatic uptake index of left lateral liver lobe; CHUI, hepatic uptake index of caudate lobe; CI, confidence interval.

## Data Availability

The datasets used and/or analysed during the current study are available from the corresponding author upon reasonable request.
